# Heart Failure Relapses in Response to Acute Stresses – Role of Immunological and Inflammatory Pathways

**DOI:** 10.3389/fcvm.2022.809935

**Published:** 2022-04-25

**Authors:** Lisa Hasselbach, Johannes Weidner, Albrecht Elsässer, Gregor Theilmeier

**Affiliations:** ^1^Division of Cardiology and Division of Perioperative Inflammation and Infection, Department Human Medicine, University of Oldenburg, Oldenburg, Germany; ^2^Division of Perioperative Inflammation and Infection, Department Human Medicine, University of Oldenburg, Oldenburg, Germany; ^3^Division of Cardiology, Department Human Medicine, University of Oldenburg, Oldenburg, Germany

**Keywords:** heart failure, inflammation, acute relapses, immunology, macrophages, infection, surgical stress response

## Abstract

Cardiovascular diseases continue to be the most imminent health care problems in the western world, accounting for numerous deaths per year. Heart failure (HF), namely the reduction of left ventricular function, is one of the major cardiovascular disease entities. It is chronically progressing with relapsing acute decompensations and an overall grave prognosis that is little different if not worse than most malignant diseases. Interestingly acute metabolically and/or immunologically challenging events like infections or major surgical procedures will cause relapses in the course of preexisting chronic heart failure, decrease the patients wellbeing and worsen myocardial function. HF itself and or its progression has been demonstrated to be driven at least in part by inflammatory pathways that are similarly turned on by infectious or non-infectious stress responses. These thus add to HF progression or relapse. TNF-α plasma levels are associated with disease severity and progression in HF. In addition, several cytokines (e.g., IL-1β, IL-6) are involved in deteriorating left ventricular function. Those observations are based on clinical studies using inhibitors of cytokines or their receptors or they stem from animal studies examining the effect of cytokine mediated inflammation on myocardial remodeling in models of heart failure. This short review summarizes the known underlying immunological processes that are shared by and drive all: chronic heart failure, select infectious diseases, and inflammatory stress responses. In conclusion the text provides a brief summary of the current development in immunomodulatory therapies for HF and their overlap with treatments of other disease entities.

## Scope of the Review

**Figure 1:**
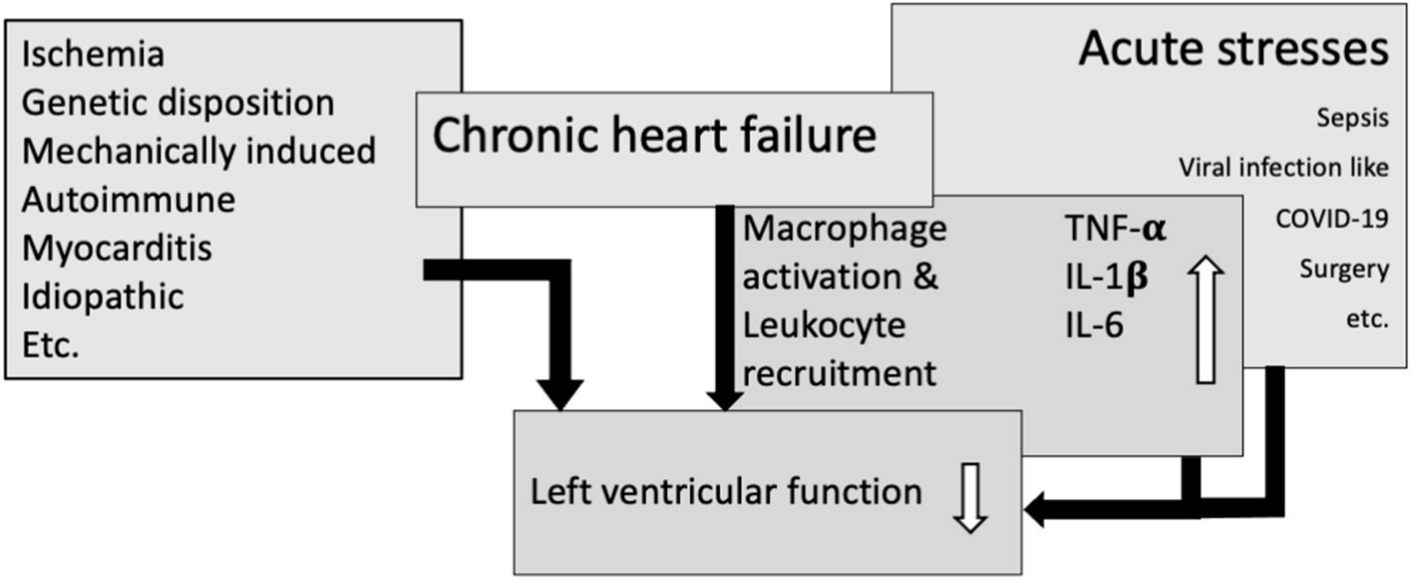
Overlapping immunological pathways in heart failure and acute stresses result in macrophage activation and leukocyte recruitment, leading to an increase in cytokines and thereby amplify impairment of left ventricular function.

Chronic heart failure (HF) is in part driven by inflammatory processes. Ventricular function in chronic HF patients worsens if the patient experiences acute illnesses or stresses. Some of these share inflammatory motifs with those active in progression of HF. We will focus on the overlap of key pathways in chronic inflammation in HF, sepsis, viral infection (i.e., COVID-19) and non-infectious stressors to shed light on their potentially synergistic adverse effects in HF progression and deterioration (Figure 1).

## Relevance of Heart Failure

HF can present as a chronic disease or in an acute setting with rapidly decreasing myocardial function either as the primary presentation or as acute decompensation of preexisting disease. Acute events (like operations or infections) can trigger a reduction of myocardial function that frequently will not return to prior levels (1).

HF has a prevalence of 11.8 % in people aged 60 or older (2). An observational study in 2,700 HF-patients showed an all-cause 5-year mortality of 53%. HF-patients over 65 years are even more likely to die (from cardiovascular—as well as non-cardiovascular causes). Hospitalization occurs about once per patient/year, mainly for non-cardiovascular causes (3).

The mainstay of HF is the loss of cardiomyocyte (functional) mass due to noxious stimuli ranging from ischemia via pressure overload-induced remodeling processes to viral infection and genetic causes (see Figure 1). Cardiomyocyte death sets off a remodeling cascade that aims to preserve left ventricular function as sufficient as possible. This remodeling cascade involves restructuring of the muscular tissue and reshuffling of its cellular and extracellular components to accommodate an optimized function in the face of the resulting new and disadvantageous ventricular geometry. The mechanisms active in this process are those of inflammatory responses, tissue breakdown, reorganization and replacement (4, 5).

### Inflammation in Heart Failure

Recently, the recognition of the role of the immune system in chronic HF is evolving (6). The most common hypothesis explaining the development of HF, is an insufficient resolution of acute inflammation resulting in chronic inflammation. Recent data suggest, that a failure in resolution of inflammation could be due to a dysregulated system of specialized pro-resolving mediators (7), leading to a peak of interest in research influencing these receptors in cardiovascular diseases (8). An interesting phenomenon in this particular context is para-inflammation, that describes a low grade inflammatory response mainly carried by tissue macrophages and perpetuated by metabolic syndrome disease entities (9, 10). Para-inflammation may chronify if it is not resolved and could thereby precipitate chronic myocardial inflammation and HF (11).

After an ischemic insult macrophages infiltrate the site of injury and alter their polarization around 1 week after the acute injury from classical proinflammatory to alternative anti-inflammatory phenotypic characteristics, the latter expressing IL-10. Inflammatory macrophages express tumor necrosis factor a (TNF-α), interleukin-6 (IL-6) and interleukin-1β (IL-1β) (12) (see Figure 1). The immune receptor responsible for production and release of these cytokines is the toll-like receptor 4 (TLR4) a receptor steering the innate immune response to tissue damage (13). Pathogen– and damage-associated molecular patterns (PAMPS and DAMPS), the ligands for TL-receptors are released from necrotic cardiomyocytes. They evoke the secretion of cytokines from resident macrophages as well as the activation and recruitment of neutrophils. TLR4 is thus actively fueling inflammation after acute myocardial injury. Its expression is regulated by cytokines, among others by IL-1β (14). TLR4 is present and upregulated in chronic HF (15).

One of the cytokines that has been shown to play an important role in HF is TNF-α since Levine et al. showed that HF patients have increased levels of circulating TNF-α (16). TNF-α- and IL-6-levels increase in episodes of acute decompensation (17). The TNF-α level correlates with worse prognosis in HF (18). *In vivo* animal experiments have shown that overexpression of TNF-α leads to chronic inflammation in the heart, to dilatative cardiomyopathy and in the end to HF (19). Blocking TNF-α showed promising results in rats reducing cardiac inflammation and adverse myocardial remodeling (20, 21).

IL-1β is upregulated in animal models of HF of different etiologies (22, 23). The knockdown of the IL-1β receptor in *in vivo* experiments prompted a reduced inflammatory response and less fibrosis after myocardial infarction in mice (24). Studies performed in animals have suggested, that IL-1β might be a good candidate molecule to influence HF. Anakinra, a receptor antagonist for IL-1β, reduced myocardial remodeling after myocardial infarction in mice (25). Targeting IL-1β with an antibody has resulted in reduced post-infarction remodeling and an improved LVEF in rats (26).

In addition are elevated levels of IL-6 associated with a increased risk of developing HF over time (27). Since IL-6 is also important in the pathophysiology of rheumatoid arthritis (RA), tocilizumab, an antibody against the IL-6 receptor has been shown to reduce pro-BNP and improve LV-function in RA patients without previously known HF (28, 29). This might imply a role for blocking IL-6 in a preventive way because patients with RA often develop HF during their course (30). Studies on the effect of blocking IL-6 in patients with known HF have not yet been conducted.

### Inflammatory Response to Non-cardiac Surgery Exemplifies the Effects of Non-infectious Stresses on Chronic Heart Failure

Surgical stress induces proinflammatory responses targeted to remove destroyed tissue, as such inducing wound healing and ultimately recovery of organ function (31–38). The prevalence of HF in the VA Surgical Quality Affairs population was 7.9% in patients undergoing non-cardiac surgery and preexisting HF resulted in increased mortality with an odd's ratio of 1.69 (CI 1.57–1.76) (39). Patients with preexisting HF undergoing non-cardiac surgery in other very large retrospective cohort studies (AHRQ-NIS) with less selected patient populations likewise carried dramatically increased mortality and rehospitalization risks (40). Preexisting HF causes rehospitalization due to CV and non-CV causes in more than 10% of the patients compared to 2.5% (41). Pre-existing chronic diseases, such as HF weaken the ability of an organism to balance pro- and anti-inflammatory responses, in short its resilience (42). In this setting, the pro-inflammatory response evoked by surgical stress may promote deterioration of left ventricular function and MI (43). Surgery induces a cytokine based inflammatory response, predominantly reflected by increases in IL-6 (38). We have shown, using a mouse model with preexisting atherosclerosis exposed to perioperative stress, that IL-6 neutralizing antibodies but not inhibition of its trans-signaling protects plaque stability, proving that IL-6 here is causal for vascular effects of surgical stress (44). In addition, surgery induces an adrenergic stress response originating from pain and tissue destruction and translating into hemodynamic changes such as increased blood pressure and heart rate. Augmented adrenergic responses will trigger more inflammation and increased blood pressure and heart rate increase the risk for deterioration of LV function (40, 42, 45).

Neutrophilia and monocytosis are hallmarks of the stress response to surgery during the first postoperative days (35–37). Neutrophils and monocytes propagate inflammatory responses targeted to remove destroyed tissue, which induces wound healing and ultimately leads to preservation of organ function (34). However, enhanced neutrophil and monocyte infiltration in inflamed myocardium may worsen HF (43, 46, 47). Hence, HF relapses after surgery may be due to elevated IL-6, monocytosis, and neutrophilia, or alternatively or in addition, hypertension and tachycardia (48).

### Heart Failure and Infection

One of the main reasons for hospitalization in patients with HF are infections. One out of four HF patients dies because of sepsis (a dysregulated host reaction of the immune system to severe infections) (49), mostly due to respiratory tract infections (50). In that context the risk of hospitalization during the influenza season is significantly increased in HF patients (51). Contracting infections on the other hand also carries a high risk to aggravate existing HF (52, 53).

There are parallels between the cytokine profile in HF patients and patients suffering from bacterial sepsis. TNF-α is elevated in septic patients (54). Survivors compared to non-survivors have significantly more TNF-α and IL-1β in their plasma (55). IL-6 levels are also elevated in sepsis and directly correlate with mortality (56). TLR4, the immune receptor responsible for the cytokine release in HF is upregulated in animal models of sepsis induced myocardial dysfunction (57), hinting at its central role in mediating what is called the “cytokine storm.”

The increased susceptibility of HF patients to infection is conveyed through several, partly independent, partly overlapping mechanisms in addition to circulating cytokines. Infections increase the metabolic demand, leading to a mismatch of oxygen demand to supply. In HF the metabolic alterations are increased oxidative stress (58), energy deficiency (59) and loss of metabolic flexibility (60) resulting in what is called sepsis-induced cardiomyopathy after infections (61). Another mainstay of sepsis is impaired renal function with volume overload and congestion that occurs particularly in HF patients. Acute kidney injury is more common in sepsis patients with preexisting HF (62).

One of the key therapeutic measures for patients in septic shock is however maintenance of intravascular volume status by infusion of fluids. Patients with preexisting HF are at high risk of acute decompensation with development of pulmonary edema due to fluid substitution. HF patients in septic shock have been shown to receive less fluids than non-HF patients. Among the HF patients those who received less fluids have a higher mortality (63). On the other hand homeostasis of intra- and extravascular fluids and electrolyte balance is important for the activation status and phenotypic characteristics of tissue macrophages. Macrophage polarity has been demonstrated to be affected by the tonicity of extravascular fluids and in turn contribute to blood pressure regulation (64, 65). If this axis is active and potentially detrimental in HF remains to be investigated.

Taken together these data suggest that it is important for HF patients to prevent infection altogether. Said that, all measures to avoid infections are effective. A very effective way to do so is vaccination. Vaccination against influenza reduces all-cause mortality and rate of hospitalization due to cardiovascular events as well as to all-causes (66–68) which has thus been recommended by international cardiology societies accordingly (69, 70).

### Heart Failure and COVID-19

Since 2020 the world has been confronted with the SARS-CoV2 pandemic. Preexisting HF increases mortality of COVID-19 (71). Exacerbation of HF can even be the only sign of a SARS-CoV2-Infection (72). Lymphocytopenia, one of the classical changes in white blood counts during COVID-19 correlates with prognosis (73), and is associated with severe HF during the early disease (74). Troponin-T showing myocardial injury, correlates with disease severity and mortality of COVID-19 even without preexisting HF (75, 76).

Like in HF and sepsis macrophage-derived cytokines are increased in COVID-19 (77, 78). Analysis of monocytes from COVID-19 patients suggests that the inflammatory response just like in HF and sepsis might be mediated through TLR4 (79). TNF-α has proven to be an independent predictor of COVID-19 severity and mortality (80).

There is contradicting data about the levels of IL-1β in COVID-19 patients. A small study showed an increase in IL-1β in COVID-19 patients correlating with the severity of the disease (81) but a larger study of roughly 1500 patients in New York has not found significant contributions of IL-1β (80).

IL-6 is significantly elevated in severe COVID-19 according to a meta-analysis and levels of IL-6 like in sepsis correlated with adverse outcomes (82). Especially the IL-6 to α1-Antitrypsin ratio is predictive for the course of the disease (81).

Post-acute sequelae of COVID-19, also called Long-COVID, is as HF potentially caused by incomplete resolution of inflammation and characterized by a long lasting elevation of IL-1β, IL-6 and TNF-α (83).

Cardiomyocytes can be directly infected with SARS-CoV2 via the angiotensin-converting enzyme type 2 (ACE2) which is then cleaved by transmembrane protease serine 2 (TMPRSS2) (84). Due to the abundant expression of ACE2 and TMPRSS2 cardiomyocytes are, apart from alveolar cells and macrophages, the main target cells for an infection with SARS-CoV2 (85). In the later course of the infection the inflammatory cytokines upregulate ACE2 resulting in a self-reinforcing process that worsens the disease (86). Because of the upregulation of ACE2 through ACE-inhibitors, an adverse effect of ACE-Inhibitors in COVID-19 has been controversially discussed (87).

ACE2 counterbalances the activated RAAS in HF by transforming Angiotensin II to angiotensin 1-7. Increased ACE2 levels thus even prevent or improve preexisting HF (88). The effect of reduced ACE2 bioavailability due to cleavage after binding SARS-CoV2 could augment RAAS-system activity and thereby blood pressure, vasoconstriction and sodium-retention, eventually leading to worsening of LV function. A small study has shown that ACE-inhibitors decrease IL-6 levels and peak viral load in COVID-19 patients (89) which has led to a change in antihypertensive management in COVID-19 patients. The optimal medical treatment for HF should be continued in COVID-Patients if the hemodynamic situation allows it (90).

Autopsies have shown that both the innate and the adaptive immune systems are activated in the heart of patients with SARS-CoV2-Infection (91). An autopsy study on 40 COVID-19 patients showed acute cardiomyocyte necrosis and apoptosis along with cardiac fibrosis in all samples. The virus itself could only be confirmed in hearts of few patients (92). Damage to the heart thus seems to be possible directly by viral infection of cardiomyocytes as well as indirectly in the course of systemic inflammatory response to local infection.

## Using Cytokine Directed Immunomodulatory Therapies to Treat Heart Sfailure

One of the cytokines indicted in inflammation in HF as well as in sepsis is TNF-α. Contrary to the promising animal experiments the use of infliximab, a TNF-α antibody resulted in a reduced cardiac output in patients with rheumatoid arthritis without known HF (93). The ATTACH trial used infliximab in patients with at least moderate HF, as well as the RENEWAL study (a combined analysis of the RENAISSANCE and RECOVER trial that used etanercept) both demonstrated that blocking TNF-α in HF patients does not yield a clinical benefit, but exerts adverse effects at higher doses (94, 95).

Numerous studies have examined the effect of monoclonal and polyclonal antibodies against TNF-α in septic patients. Both reduce over-all mortality in patients with severe sepsis. Monoclonal anti-TNF-α-antibodies improve survival of patients in septic shock (96). In COVID-19 the use of adalimumab, a TNF-α-antibody did not reduce mortality or beneficially alter the course of the disease (97).

These results suggest that even though TNF-α is upregulated in HF as well as in other infections, blocking it by using antibodies has negative effects on HF despite positive effects on some select infectious diseases.

In the CANTOS-trial canakinumab, an IL-1β antibody, in patients with myocardial infarction and active inflammation resulted in less all-cause mortality and a lower rate for HF hospitalization (98, 99). Also canakinumab did not result in a reduction of COVID-19 mortality (100). Anakinra, the IL-1β receptor antagonist which had proven effective in reducing adverse myocardial remodeling in mice also successfully improved left ventricular function after myocardial infarction in humans (101). It also reduced mortality in septic patients (102). In the REMAP-CAP trial anakinra did not have a positive effect on COVID-19 (103).

A positive effect of the anti-IL-6 antibody tocilizumab has been seen for the cardiac function of patients without HF (28, 29). Up to now no studies have been performed on the use of tocilizumab or other IL-6 Inhibitors in HF patients but a case report of a patient with HF after myocardial infarction who received tocilizumab because of a large vessel arteriitis has shown an improvement of myocardial function after the treatment (104). In the IMICA trial tocilizumab has reduced inflammation as well as myocardial damage in post-cardiac arrest syndrome (105). After a series of cases and small studies of a successful treatment of COVID-19 related HF and cytokine storm with tocilizumab had been reported (106, 107), the REMAP-CAP trial has evaluated the effect of the IL-6 receptor antibodies tocilizumab and sarilumab (103). There is now a recommendation to use tocilizumab or sarilumab in patients with severe COVID-19 without invasive ventilation (108). Whether the effect of anti-IL-6 receptor antibodies is due to the blocking of myocardial IL-6 receptors or other effector organs has still to be examined. In sepsis no large scale studies have been performed on blocking IL-6 but tocilizumab has proven to attenuate infection and improve survival in an animal model of sepsis (109). Of the examined cytokines IL-6 seems to offer the most potential for immunomodulatory therapy to affect both infections and HF.

In a broader attempt to address the immunological aspects in HF the ACCLAIM study showed better over-all survival in the mildly impaired (NYHA II) group after using a non-specific immunomodulatory therapy (using the Celacade system™) (110). Surprisingly, follow-up studies revealed that the circulating antibody amount is the same with or without receiving non-specific immunemodulation (111).

CytoResc, a recent, not yet published, trial uses an extracorporeal polystyrene-based hemoadsorber in COVID-19 patients (112). Prior to this study, a single-center study was performed in Freiburg using extracorporeal membrane oxygenation in combination with hemoadsorption, leading to no reduction in IL-6 and even decreased survival (113). Interestingly, even though resulting in clinically favorable outcome in septic patients (114) said hemoadsorber did not change the levels of IL-1β, IL-6 or TNF-α (115).

The use of corticosteroids is a treatment option for patients in septic shock if treatment with vasopressors and fluids is not successful in improving the hemodynamic situation (116). For a long time, using an unspecific immunosuppression with corticosteroids was the only effective treatment for COVID-19 patients (117). In HF use of corticosteroids is debated controversially: While it may decrease chronic inflammation it also affects the neurohormonal RAA system by inducing ACE (118). Modulation of the RAAS by using ACE-Inhibitors or angiotensin receptor neprilysin inhibitors (ANRI) is one of the main therapeutic components in HF. It reduces mortality and morbidity with the ANRI valsartan in combination with sacubitril showing superiority over Enalapril in the PARADIGM-HF study (119, 120). However, the CORT-AHF study did not show a difference in adverse events for patients with or without HF after corticosteroid treatment, this result was independent from use of ACE-Inhibitors or ANRI (121).

## Conclusion

The aim of this short review was to delineate parallels in the immunological processes in HF and inflammatory responses to infections or non-infectious stresses, which can also be viewed as an opportunity for using immune based strategies to fight deteriorating HF in cases with such comorbidities.

Influencing the release and circulation of cytokines could be an interesting starting point for HF therapy but is not yet ready to be administered in a broad setting. Inhibition of IL-6-signaling seems to be one of the more promising strategies for treatment of HF as well as infections but it has to be considered, that there is a lot of contradictory data so that a clear recommendation for immunomodulatory therapy cannot be given yet.

TLR4 is involved in the development of various diseases connected to inflammation is. To date TLR4 signaling cannot be therapeutically targeted, while this may be a promising avenue to improve cardiac function in HF patients as well as to reduce myocardial dysfunction in infectious diseases (122).

One thing one should keep in mind while discussing immunomodulatory therapy is that these medications have a risk of increasing susceptibility for new infections necessitating a strict indication and close monitoring during and after application.

## Author Contributions

The manuscript was written by LH and GT and revised by JW and AE. All authors contributed to the article and approved the submitted version.

## Funding

Funding for LH was provided through the University of Oldenburg (Forschungspool-Projekt 2018-030).

## Conflict of Interest

The authors declare that the research was conducted in the absence of any commercial or financial relationships that could be construed as a potential conflict of interest.

## Publisher's Note

All claims expressed in this article are solely those of the authors and do not necessarily represent those of their affiliated organizations, or those of the publisher, the editors and the reviewers. Any product that may be evaluated in this article, or claim that may be made by its manufacturer, is not guaranteed or endorsed by the publisher.
